# Effect of Acupuncture for Mixed Urinary Incontinence in Women: A Systematic Review

**DOI:** 10.3389/fpubh.2022.827853

**Published:** 2022-03-18

**Authors:** Zilin Long, Huan Chen, Shudan Yu, Xinlu Wang, Zhishun Liu

**Affiliations:** ^1^Department of Acupuncture, Guang'anmen Hospital, China Academy of Chinese Medical Sciences, Beijing, China; ^2^Graduate School, Beijing University of Chinese Medicine, Beijing, China; ^3^Graduate School, Tianjin University of Traditional Chinese Medicine, Tianjin, China

**Keywords:** mixed urinary incontinence, women, systematic review, acupuncture, effect

## Abstract

**Background:**

Mixed urinary incontinence increasingly undermines women's quality of life. Previous studies showed some effects of acupuncture for MUI, but no systematic review has been done to evaluate the efficacy and safety of acupuncture for MUI in women.

**Objective:**

To systematically review the efficacy and safety of acupuncture for women with MUI.

**Methods:**

Ten databases (i.e., PubMed, Web of Science, Embase, ClinicalTrials.gov, the Cochrane Library, CBM, Scoups, CNKI, VIP and WANFANG DATA) were searched up to July 19th, 2021, using tailored search strategies with keywords not limited to “female,” “mixed urinary incontinence,” “acupuncture,” and “randomized controlled trial,” etc. RCTs and quasi-RCTs were included if investigating effect of any type of acupuncture for female patients with MUI. Data were extracted from eligible studies, and risks of bias were assessed according to the Cochrane Handbook from seven aspects using the RevMan 5.4 software.

**Results:**

A total of three randomized studies with 591 women were included. The risk of bias among the studies varied, with major concerns on blinding of participants and outcome assessor. Liu's study (497) mainly showed that electroacupuncture's effect on reduction of numbers of incontinence, urgency, nocturia episodes, and amount of urine leakage etc. was not inferior to that of PFMT-Solifenacin group at 12, 24, and 36 weeks. Zhan's study (60) showed that electroacupuncture reduced significantly more amount of urine leakage than Tolterodine at 8 weeks, with no data on incontinence episodes frequency. All 3 studies reported that acupuncture significantly increased the quality of life assessed by ICIQ score. In addition, incidence of acupuncture-related adverse events was rare, while antimuscarinic agents related adverse events were common in two studies.

**Conclusion:**

Although acupuncture showed some benefit for women with MUI, more evidences were required to draw a solid conclusion of effectiveness and safety of acupuncture for women with MUI.

**Systematic Review Registration:**

https://www.crd.york.ac.uk/PROSPERO, identifier: CRD42021224600.

## Introduction

Mixed urinary incontinence (MUI), as a type of urinary incontinence (UI), presents with involuntary leakage associated with urgency and also with exertion, effort, sneezing or coughing, which is more bothersome than either stress or urgency incontinence alone (1–3). MUI was predicted to affect more women than men (4) and its epidemiological data varied widely. For example, prevalence rates of MUI among women were 0.7–26.6, 15.7–36.6, 9–18, and 40% in China, United State, Egypt, and England, respectively (5–8).

Pathophysiological mechanisms of MUI are unclear (9). It might be a combination of the mechanisms of stress urinary incontinence (SUI) and urgency urinary incontinence (UUI), namely, intrinsic urethral sphincter deficiency, urethral hypermobility, detrusor overactivity, or combinations of above factors (10). Although MUI is less common than stress urinary incontinence, previous studies have shown that women with MUI may have lower level of quality-of-life than those with SUI or UUI alone (11).

Nowadays pelvic floor muscle training (PFMT) is recommended as the first-line therapy for MUI (12), even if it focuses more on the stress component and requires 3–6 months to reach its full effect (1). Its success depends on the patient's knowledge of the training program and adherence (13). Pharmacological managements including antimuscarinic agents, Beta 3-adrenergic agonists and Duloxetine are recommended as the second-line therapy (2, 14), however, the former two medicines are only effectively for the urgency component of MUI rather than the stress component (15, 16), while Duloxetine is on the contrary (17). Surgery is considered only when stress urinary incontinence is the pre-dominant component of MUI and failed with conservative treatments (2), and yet surgery might also worsen the symptoms of urgency for patients with MUI (18). Hence, management of mixed incontinence is very challenging due to poor response to current therapeutic approaches, and lacking of treatment tackling both stress and urgency symptoms of MUI (2).

Acupuncture is widely accepted as an alternative treatment. Existing studies (19–21) show that acupuncture can improve symptoms for patients with urinary incontinence, but they focused on pure SUI or pure UUI. There were no systematic reviews evaluating the effectiveness and safety of acupuncture for female with MUI. Therefore, we conducted this systematic review to investigate whether acupuncture has effect on MUI among female patients.

## Methods

The systematic review was conducted in the light of the Cochrane Handbook for Systematic Review of Interventions (22) and was registered at the National Institute for Health Research PROSPERO, International Prospective Register of Systematic Reviews https://www.crd.york.ac.uk/PROSPERO, Registration Number: CRD42021224600.

### Literature Research

Ten databases including PubMed, Web of Science, Embase, ClinicalTrials.gov, the Cochrane Library-Trials, Scoups, CBM, CNKI, VIP and WANFANG DATA were searched from inception up to July 2021. The keywords included “female,” “mixed urinary incontinence,” “urinary incontinence,” “acupuncture,” “electroacupuncture,” “scalp acupuncture,” “auricular acupuncture,” “intradermal acupuncture,” “abdominal acupuncture,” “dry needle,” “fire needle,” or “elongated needle,” “randomized controlled trial,” “quasi-randomized controlled trial,” “RCTs,” etc. The tailored search strategy was developed for each database, and the search was completed on July 19th, 2021 (refer to Appendix A for detailed search strategies).

### Inclusion and Exclusion Criteria

Studies would be included if they were (1) investigating adult female patients diagnosed with MUI: the symptoms should combine both SUI and UUI symptoms; (2) randomized controlled trials (RCTs) or quasi-RCTs; (3) comparing any type of acupunctures (including manual acupuncture (MA), electroacupuncture (EA), scalp acupuncture, auricular acupuncture, intradermal acupuncture, abdominal acupuncture, dry needle, fire needle or elongated needle) with surgery, sham acupuncture, medicine, any other non-surgical therapies, or no treatment; (4) evaluating outcome variables on amount of urine leakage in 1 h, urinary incontinence episodes, micturition frequency, and nocturia episodes in 24 h, severity of urinary urgency, quality of life, and etc. No limit was placed for time of publication and language.

Studies would be excluded if they were (1) patients with pure SUI or pure UUI; (2) comparing one type of acupuncture with another type of acupuncture; (3) case report or series, cross-sectional studies, self-controlled studies, case-control studies, cohort studies, other observational studies or laboratory experiments; (4) reviews, protocols, secondary analysis, conference abstracts or posters; (5) not providing outcome data or information.

### Study Selection and Data Extraction

All the search results were downloaded and managed by the EndNote (version X8). Two independent reviewers (ZL and HC) reviewed titles and abstracts of all retrieved articles according to the inclusion and exclusion criteria. Then full texts of articles were retrieved from above mentioned databases where necessary and reviewed carefully by the two reviewers (ZL and HC) to confirm eligible studies, and any disagreement was resolved by the supervisor (ZL).

When studies had multiple publications, the one reporting the latest or complete outcome data was included. Another two researchers (XW and SY) extracted data from the original publications, including authors' name, country, year of publication, study design, sample size, patient's mean age, treatment type and regimen of experiment and control group, frequency and duration of treatments, follow-up time, outcome measures and results, and adverse events.

### Assessment of Risk of Bias

Risk of bias of each study included was assessed according to the Cochrane Handbook from seven aspects, including random sequence generation, allocation concealment, blinding of participants and personnel, blinding of outcome assessor, incomplete outcome data, selective reporting and other bias by using software- Review Manager 5.4 (22). Two independent researchers (ZL and HC) assessed included studies for risk of bias, and any disagreement was resolved by discussion.

### Data Analysis and Synthesis

For continuous outcome variables, the mean difference (MD) with 95% confidence intervals (CIs) was used to present treatment effect between treatment groups. For dichotomous variables, risk ratio (RR) with 95% CIs was adopted to report difference on treatment effects.

Meta-analysis would be undertaken to synthesize outcome variables using dedicated software-Review Manager 5.4 only if the interventions were homogeneous based on clinical criteria. Whereas, qualitative analysis would be done if high heterogeneity was found among included studies.

## Results

### Literature Search and Study Selection

**Figure 1:**
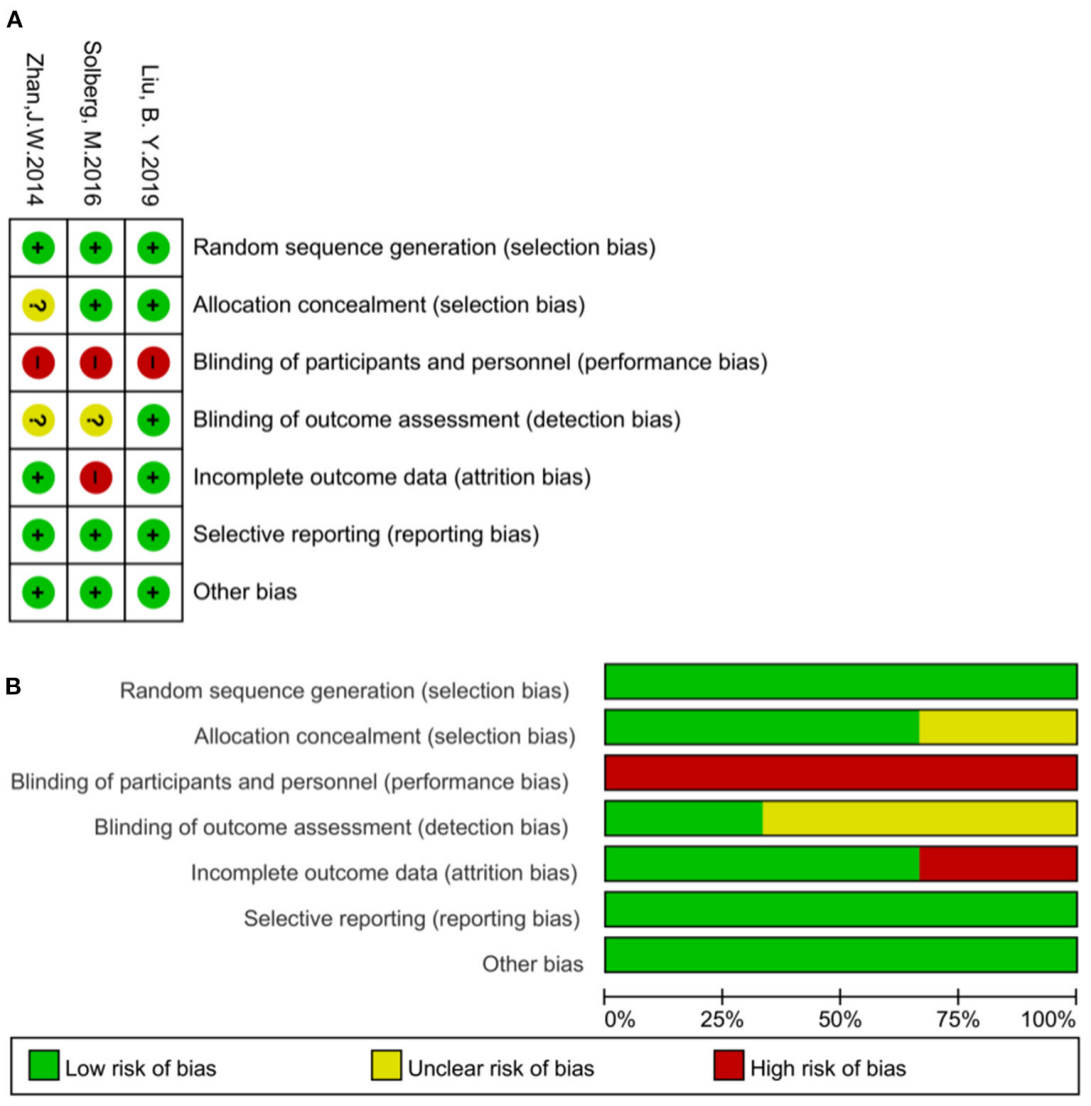
**(A)** Risk of bias summary: review authors' judgements about each risk of bias item for each included study. **(B)** Risk of bias graph: review authors' judgements about each risk of bias item presented as percentages for all included studies.

There were 157 studies identified from ten databases, out of which 25 were reviewed in full text. Two studies were excluded as independent data on MUI were not available (23, 24). Eventually three RCTs were eligible and included in the analysis. The flowchart of selection process of studies was shown in Figure 1.

### Characteristics of the Included Studies

The three included trials enrolled 591 women with MUI. Liu et al.'s study described MUI type of patients (48.3% stress-predominate, 35.8% urge-predominate, 15.9% balanced) (25), while the other two studies (26, 27) did not distinguish MUI type of patients. Substantial variation was found on design of study and control group among included studies, of which one compared EA with Tolterodine (26), one compared MA with PFMT and waiting, respectively (27), and one compared EA with solifenacin plus PFMT (25). Apart from duration of single treatment session (30 min on average), high heterogeneity was found among treatment regimen in acupuncture group across studies, including type of acupuncture (EA and MA), frequency and total duration of treatment, acupoints selected, intensity and frequency of electric current of EA, etc (Table 1).

**Table 1:** Characteristics of studies included in the review.

**Author (year,** **country,** **design)**	**Population**		**Sample Size** **(drop-outs)**			**Age (years**, **mean ± SD)**			**Intervention**			**Follow-up** **(weeks)**		**Outcome**
	**T**	**C**	**T**		**C**	**T**		**C**	**T**		**C**	**T**	**C**	
Zhan (2014, China, RCT)	FMUI		30 (0)		30 (0)		Not reported		Electroacupuncture 1. Acupoints: BL33, BL35, ST36, SP6. 2. BL33, BL35: a depth of 50 to 60 mm. 3. 30-min/time at 10/50 Hz, 0.1–5.0 mA 4. 3-time/week for 8 weeks.		Tolterodine 2 mg orally, one tablet, once a day for 8 weeks	None		1. Urine leakage measured by pad test (g)/24 h 2. ICIQ-SF
Solberg (2016, Norway, RCT)	FMUI		12 (4)	10 (4)	12 (6)	60.75 ± 14.98	63.63 ± 15.20	52.50 ± 14.37	Manual acupuncture 1. Acupoints: CV3, CV4, CV6, SP6, KI3, KI7, BL31-34, BL23, BL28, GV4, GV20 2. 30-min/time 3. 12 times in 12 weeks.	Pelvic floor muscle training (PFMT) 1. One individual consultation with A specialist trained female physio-therapist before PFMT 2. Writing an exercise diary every day 3.10-min/time, daily for 12 weeks	No treatment	12		1. ICIQ-SF 2. Adverse event
Liu (2019, China, RCT)	FMUI (Stress-dominant: 53.01% Urge-dominant: 33.33% Balanced: 13.65%)	FMUI (Stress-dominant: 43.55% Urge-dominant: 38.31% Balanced: 18.15%)	249 (4)		248 (13)	54.70 ± 10.01		53.70 ± 9.40	Electroacupuncture 1. Acupoints: BL33, BL35. 2. A depth of 50 to 60 mm. 3. 30-min at 10/50 Hz, 0.1–5.0 mA. 4.3-time/week for 12 weeks.		Solifenacin + pelvic floor muscle training 1. 5 mg orally, once a day for 36 weeks 2. Intensive exercises (1–12 wks: once/week; 13–36 wks: once/month) + homebased exercise (3 times/day) for 36 weeks	24	None	1. Percentage change in 72 h IEF 2. Reduction ≥ 50% in 72 h-IEF 3. Urgency/urination/ nocturia episodes /72 h 4. Urine leakage measured by the 1 h pad test (g) 5. Weekly mean no. of urine pads used 6. No. of participants using urine pads 7. ICIQ-SF 8. Patient satisfaction 9. Improvement degree 10. Adverse event

*T, treatment group; C, control group; SD, standard deviation; FMUI, female with mixed urinary incontinence; RCT, randomized control trial; ICIQ-SF, International Consultation on Incontinence Questionnaire-Short Form; IEF, incontinence episode frequency*.

In terms of outcome variables, amount of urinary leakage was assessed in Liu et al.'s and Zhan et al.'s study by pad test (25, 26). Number of incontinence episode, micturition, nocturia episodes, urgency and patients using urine pads, as well as improvement degree by the Patient Global Impression of Improvement (PGI-I) and patient satisfaction, were only evaluated in Liu et al.'s study using 72-h bladder voiding diary (25). Quality of life was assessed in all three studies (25–27) by the International Consultation Incontinence Questionnaire Short Form (ICIQ-SF). Adverse events were reported by Liu et al. and Solberg et al.'s studies (25, 27). Table 2 summarized the outcomes of included studies.

**Table 2:** Summary of outcomes.

**References**	**Outcome measured**		**Experiment group**			**Control group**			**Difference of changes**	
			**Baseline**	**After treatment**	**Changes**	**Baseline**	**After treatment**	**Changes**	MD or RR (95% CI)^*^	*P*-value^*^
Zhan et al. (26)	Urine leakage measured by pad test (g)/24 h (Mean ± SD)		18.27 ± 4.68	16.17 ± 4.94	−2.10 ± 2.26	15.61 ± 3.43	14.97 ± 3.84	−0.64 ± 0.97	−1.46 (−2.34, −0.58)	0.001
	ICIQ-SF (Mean ± SD)		16.30 ± 1.80	9.21 ± 3.34	−7.09 ± 3.00	14.42 ± 2.08	11.25 ± 3.50	−3.17 ± 2.73	−3.92 (−5.37, −2.47)	<0.001
Solberg et al. (27)	ICIQ-SF (Mean ± SD)		11.00 ± 3.06	5.97 ± 3.66	−5.03 ± 1.57	7.25 ± 2.26 (PFMT)	6.37 ± 3.31	−0.88 ± 2.93 (PFMT)	−4.15 (−6.73, −1.57)	0.002
						10.75 ± 3.06 (waiting)	9.62 ± 3.51	−1.13 ± 1.76 (waiting)	−3.90 (−5.68, −2.12)	<0.001
	Adverse events [*n* (%)]			25.00%(2/8)			16.67%(1/6) (PFMT)		1.50 (0.71, 12.94)	0.71
							0.00%(0/6) (waiting)		3.89 (0.22, 68.67)	0.35
Liu et al. (25)	Percentage change in 72 h IEF	1–12 wks			−37.83 ± 33.25			−36.49 ± 59.30	−1.34 (−9.78, 7.10)	<0.001
		13–24 wks			−58.20 ± 40.13			−56.69 ± 40.82	−1.52 (−8.63, 5.6)	0.68
		25–36 wks			−64.20 ± 36.79			−65.48 ± 37.44	1.28 (−5.24, 7.81)	0.70
	Reduction ≥ 50% in 72 h-IEF [*n* (%)]	1–12 wks		109/248 (44.00%)			112/239 (46.90%)		0.94 (0.77, 1.14)	0.52
		13–24 wks		174/245 (71.00%)			164/235 (69.80%)		1.02 (0.91, 1.14)	0.77
		25–36 wks		190/245 (77.60%)			189/235 (80.00%)		0.96 (0.88, 1.06)	0.44
	Urgency episodes/72 h (Mean ± SD)	1–12 wks	8.4 ± 9.4	5.78 ± 11.40	−2.62 ± 4.63	8.5 ± 8.3	5.51 ± 9.59	−2.99 ± 4.70	3.70 (−0.45, 1.19)	0.38
		13–24 wks		3.65 ± 11.45	−4.75 ± 4.55		3.73 ± 9.62	−4.77 ± 4.66	0.02 (−0.79, 0.83)	0.96
		25–36 wks		2.85 ± 11.40	−5.55 ± 4.63		2.55 ± 9.55	−5.55 ± 4.74	0.00 (−0.82, 0.82)	1
	Urine leakage measured by pad test (g)/h (Mean ± SD)	Week 4	19.70 ± 25.70	12.96 ± 32.96	−6.74 ± 9.54	18.9 ± 19.80	11.94 ± 24.05	−6.96 ± 9.68	0.22 (−1.47, 1.91)	0.8
		Week 12		7.54 ± 33.55	−12.16 ± 8.17		7.41 ± 24.92	−11.49 ± 8.28	−0.67 (−2.12, 0.78)	0.36
	Weekly mean use of urine pads (Mean ± SD)	1–12 wks	8.50 ± 1.33	8.00 ± 1.00	−0.50 ± 1.20	9.00 ± 1.50	8.75 ± 1.67	−0.25 ± 1.60	−0.25 (−0.50, 0.00)	0.05
		13–24 wks		7.00 ± 1.00	−1.50 ± 1.20		8.25 ± 1.67	−0.75 ± 1.60	−0.75 (−1, −0.50)	<0.001
		25–36 wks		7.5 ± 1.00	−1.00 ± 1.20		8.25 ± 1.67	−0.75 ± 1.60	−0.25 (−0.50, 0.00)	0.05
	No. of participants using urine pads [*n* (%)]	1–12 wks	136/249 (54.62%)	86/248 (34.68%)		150/248 (60.48%)	105/240 (43.75%)		0.57 (0.47, 0.70)	<0.001
		13–24 wks		68/244 (27.87%)			80/237 (34.60%)		0.83 (0.63, 1.08)	0.16
		25–36 wks		59/244 (24.1%)			72/237 (30.38%)		0.78 (0.59, 1.07)	0.13
	IEF/72h (mean ± SD)	1–12 wks	11.90 ± 9.90	7.34 ± 12.17	−4.56 ± 4.63	11.70 ± 9.80	7.33 ± 11.99	−4.37 ± 4.66	−0.19 (−1.03, 0.65)	0.66
		13–24 wks		4.63 ± 12.22	−7.27 ± 4.55		4.75 ± 12.02	−6.95 ± 4.62	−0.32 (−1.13, 0.49)	0.44
		25–36 wks		3.59 ± 12.41	−8.31 ± 4.23		3.48 ± 12.22	−8.22 ± 4.30	−0.09 (−0.84, 0.66)	0.81
	Urination episodes /72 h (mean ± SD)	1–12 wks	31.20 ± 11.90	27.97 ± 14.58	−3.23 ± 5.64	29.90 ± 9.40	26.28 ± 10.51	−3.62 ± 5.70	0.39 (−0.61, 1.39)	0.44
		13–24 wks		25.24 ± 14.36	−5.96 ± 5.96		24.28 ± 10.12	−5.62 ± 6.07	−0.34 (−1.40, 0.72)	0.53
		25–36 wks		24.18 ± 13.8	−7.02 ± 6.68		23.79 ± 9.13	−6.11 ± 6.83	−0.91 (−2.10, 0.28)	0.13
	Nocturia episodes/72 h (mean ± SD)	1–12 wks	3.90 ± 3.00	3.11 ± 3.10	−0.79 ± 2.05	3.80 ± 3.10	2.93 ± 3.57	−0.87 ± 1.77	0.08 (−0.26, 0.42)	0.64
		13–24 wks		2.52 ± 3.36	−1.38 ± 1.81		2.47 ± 3.53	−1.33 ± 1.81	−0.05 (−0.37, 0.27)	0.76
		25–36 wks		2.32 ± 3.44	−1.58 ± 1.73		2.22 ± 3.57	−1.58 ± 1.77	0.00 (−0.31, 0.31)	1
	ICIQ-SF (mean ± SD)	1–12 wks	12.70 ± 2.50	8.74 ± 3.12	−3.96 ± 2.86	12.90 ± 2.20	9.17 ± 3.29	−3.73 ± 2.90	−0.23 (−0.74, 0.28)	0.37
		13–24 wks		6.26 ± 4.49	−6.44 ± 3.90		6.93 ± 4.36	−5.97 ± 3.78	−0.47 (−1.15, 0.21)	0.17
		25–36 wks		5.64 ± 4.20	−7.06 ± 3.66		6.1 ± 4.27	−6.80 ± 3.70	−0.26 (−0.91, 0.39)	0.43
	Patient satisfaction^a^ [*n* (%)]	Week 12		71.60% (174/243)			60.68% (142/234)		1.18 (1.04, 1.34)	0.01
		Week 36		76.23% (186/244)			71.37% (167/234)		1.07 (0.96, 1.19)	0.23
	Improvement degree^b^	Week 12		63.11% (154/244)			54.27% (127/234)		1.16 (1.00, 1.35)	0.05
		Week 36		73.47% (180/245)			70.64% (166/235)		1.15 (0.77, 1.72)	0.49
	Adverse events [*n* (%)]			41/249 (16.47%)			91/248 (36.69%)		0.45 (0.32, 0.62)	<0.001

*MD, mean difference; RR, Relative Risk; SD, standard deviation; IEF, incontinence episode frequency; ICIQ-SF, International Consultation on Incontinence Questionnaire-Short Form.*

* *MD, RR, and P-value were calculated based on data provided in the original papers using Revman V5.4. MD was calculated as mean difference of treatment effect (post-treatment value minus baseline value) in each group.*

a *Satisfaction with treatment outcome measured on a 5-point Likert scale (marked dissatisfaction to marked satisfaction).*

b *Improvement degree measured by Participant Global Impression Improvement on a 7-point Likert scale (marked worsening to marked improvement)*.

### Assessment of Risk of Bias

**Figure 2:**
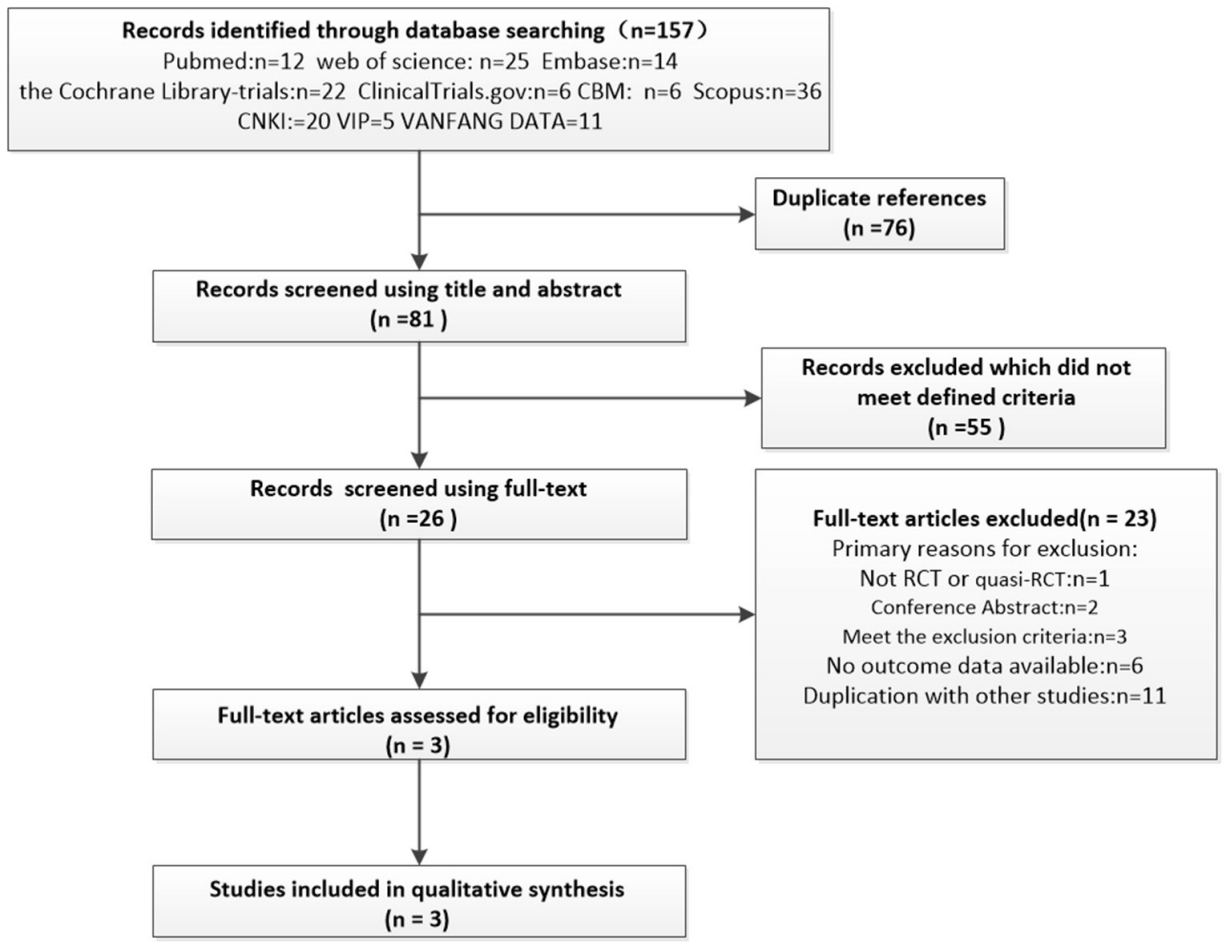
The flowchart of the selected studies.

Among the three studies included in this review, all had random sequence generation, Liu et al. and Solberg et al. reported concealment of allocation (25, 27), Zhan et al.'s study (26) did not report how allocation concealment was done. None of the studies (25–27) blinded subjects in consequence of open-label trials which compared acupuncture with drugs, PFMT or no intervention, while Liu et al.'s study (25) reported blinding of outcome evaluators. Solberg et al.'s study (27) had high risk of attrition bias as its dropout rate was >40% and did not report how the missing data was dealt with. There was no reporting bias or other bias identified in any of the studies. Risk of bias assessment is presented in Figure 2.

### Effect of Acupuncture

#### EA vs. PFMT and Solifenacin

Liu et al.'s study (25) reported a multicenter, randomized controlled, non-inferiority trial to compare the efficacy and safety of EA with a combination of PFMT and solifenacin for women with MUI.

The study reported that the percentage of reduction from baseline in mean 72-h incontinence episode frequency (IEF) over weeks 1–12 (primary outcome) was comparable in EA and PFMT-solifenacin group (37.83 vs. 36.49%, *P* > 0.05). Non-inferiority for the percentage of reduction from baseline in mean 72-h IEF in EA group was sustained up to 36 weeks since initiation of the treatment.

Change in amount of urine leakage measured by 1-h pad test, as recommended by the International Continence Society (28), was not significantly different between the two groups at week 4 (*P* = 0.8) and week 12 (*P* = 0.36). The proportions of participants with at least 50% reduction in the mean 72-h IEF were also comparable between the two groups weeks 1–12 (*P* = 0.77), weeks 13–24 (*P* = 0.52), and weeks 25–36 (*P* = 0.44), respectively.

Improvements were observed on all other secondary outcome measures in both groups, including change in 72-h urgency/incontinence/nocturia episodes, number of participants using urine pads and weekly mean use of urine pads, and no significant difference between groups was found at any of the follow-up assessments except for the number of participants using urine pads during weeks 1–12 (*P* < 0.001).

The study reported that the changes in the ICIQ-SF score were similar in EA and PFMT-solifenacin groups at weeks 12, 24, and 36 without statistical differences (*P* = 0.37, *P* = 0.17, *P* = 0.43, respectively). Notably, the decline of ICIQ-SF score in EA group were higher than the minimum clinically important difference (MCID) of 4 (29).

In addition, the EA group had better satisfaction (satisfaction and marked satisfaction) rates compared to PFMT-solifenacin group at week 12 (71.6 vs. 60.68%) with a statistical difference (*P* = 0.01).

Total incidence of adverse events in EA group was significantly lower than that in PFMT-solifenacin group (16.47 vs. 36.69%, *P* < 0.001). Four percent of patients in EA group reported EA related mild subcutaneous hematoma and did not require any treatment, while, 28.22% of patients in PFMT-solifenacin group reported digestive system symptoms, especially dry mouth (25%), which led to poor tolerance and patient compliance.

#### EA vs. Tolterodine

Zhan et al.'s study (26) reported a single-center RCT of 60 women with MUI to investigate the clinical effect of EA in comparison with Tolterodine. The study adopted similar treatment regimen to Liu et al.'s study (25) in terms of frequency of treatment (3-time per week) and electric current for EA (10/50 Hz), but with two more acupoints (ST36, SP6) and less total treatment duration (8 weeks).

The study adopted 24 h-pad test to evaluate the change on amount of urine leakage, which provides more information for the estimation of the incontinence severity (30), and ICIQ-SF score for quality of life. It was found that the patients in EA group had more reduction from baseline on among of urine leakage compared with that in tolterodine group (−2.1 ± 2.26 vs. −0.64 ± 0.97, *P* = 0.001), and the change on ICIQ-SF score from baseline in EA group was significantly more than that in tolterodine groups at week 8 (−7.09 ± 3.00 vs. −3.17 ± 2.73, *P* < 0.001). However, neither outcome data at longer follow up time, nor any kinds of adverse event was reported in this study. Additionally, it only mentioned the random number table for patient's allocation without any description on allocation concealment.

#### Acupuncture vs. PFMT vs. Waiting

Solberg et al. (27) conducted a three-arm RCT to examine the efficacy of acupuncture in comparison with PFMT and waiting groups among 34 women with MUI in a 12-week period. The manual acupuncture (30-min per time) was applied for 12 times in 12 weeks, and PFMT (10-min per time) was conducted once a day for 12 weeks. However, only 20 women completed the trial in total, with 8 in MA group, 6 in PFMT group and 6 in waiting group, and the dropout rates were as high as 25% in MA group, 40% in PFMT group, and 50% in waiting group, respectively.

The study showed that the reduction of ICIQ-SF score from baseline in MA group was significantly more than that in either PFMT (−5.03 ± 1.57 vs. −0.88 ± 2.93, *P* = 0.002) or waiting (−5.03 ± 1.57 vs. 1.13 ± 1.76, *P* < 0.001) groups at week 12. However, no data on amount of urine leakage, numbers of incontinence episodes or micturition etc was reported, nor did the long-term effect of treatment in either group. Two women experienced adverse events (one with fatigue and one with worsened incontinence after the initial few therapies) in acupuncture group, one woman reported worsened incontinence in PFMT group at the beginning.

## Discussion

Two included studies showed that EA could reduce the amount of urine leakage. One study with sound quality and large sample size showed that the effect of EA was not inferior to PFMT-solifenacin on improvement of incontinence, and another study with relatively small sample size reported that EA showed more benefit on reduction of involuntary loss of urine than Tolterodine. All three included studies indicated EA/MA could improve patient's quality of life. However, the risk of bias among the three studies varied, with major concerns on risk of blinding of participants and outcome assessor. And small sample size (26, 27) and high drop-out rate (27) were found in two third of included studies made it even more challenging to draw a conclusion on the effect of acupuncture on MUI.

As we know, the underlying mechanism of acupuncture on urinary incontinence has been discussed in a number of previous studies, which showed that sacral, pudendal and pelvic nerve had a vital role in pathogenesis and treatment of urinary continence (31). Acupuncture on the lumbosacral points may strengthen the function of sympathetic and pudendal nerves, suppressing excessive contractions and overactivity of detrusor, and improving bladder compliance to treat urgent incontinence (32). In addition, it may also promote the contraction of the pelvic floor muscle, increasing the stability of the pelvic floor structure to relieve the symptom of stress incontinence (33). However, rigorous evidence to proof above mechanisms and effect of acupuncture on MUI is not yet adequate to guide clinical practice, compared with evidences on acupuncture for stress urinary alone (34).

As found in this review, studies tried to provide evidences on acupuncture's effect using diverse outcome measurements. As a recommended measurement to quantify the amount of urine leakage for patient with UI, the pad test was often used with a length of 1- or 24-h (35). In this review, two studies reported that the effect of EA was equivalent or better than that of interventions of control groups by comparing changes on amount of urine leakage between the two groups (25, 26). However, the pad test was not able to provide specific information to help researchers to trace whether the decreased amount of urine leakage was from improvement of stress or urgency symptoms or both after acupuncture treatment (36). In contrast, the 72-h voiding diary, another recommended approach, could separately quantify the change on stress and urgent incontinence by recording the numbers of each type of urinary incontinence (37). With such measurement, Liu et al.'s study added further evidences in favor of EA on MUI at the completion of treatment at 12 weeks and longer follow-up at 36 weeks, in comparison to active treatments (PFMT and Solifenacine) (25). In addition, all 3 included studies (25–27) provided data on quality of life, and showed that both EA and MA could generate clinically significant improvement on quality of life measured by ICIQ-SF scores with a MCID of 4 (29). Such results were consistent with previous systematic review of 15 studies (38). However, given the high heterogeneity found among population (severity of symptoms etc.), study design (especially design of control group and statistical methods used), treatment regimens (i.e., the selection of acupoints, intensity of electric current, frequency and length of treatment) and outcome measures, as well as inadequate number of studies included, it was unlikely to conduct a meta-analysis to estimate the pooled effect size.

In terms of the safety of acupuncture, two studies reported adverse events (25, 27), and found that the incidence rate of adverse events of acupuncture was comparable with PFMT alone (27), but significantly lower than that of PFMT-solifenacin (25). It was well known that the frequent occurrence of adverse events of antimuscarinic agents, such as dry mouth and constipation, can lead to patients' poor compliance and thus reduce treatment effect (39), either used alone or combined with other treatments. For safety consideration, novel and alternative treatments for urgent or mix incontinence is worth exploring and more evidences are required.

It is also worth mentioning that none of the studies used placebo/sham acupuncture as control group to evaluate the net effect size of acupuncture. Although Solberg's trial (27) compared the effect of acupuncture with no treatment (waiting for acupuncture or PFMT), the small sample size (34 participants) and a high drop-out rate (more than 40%) undermined the reliability of its conclusion. This has shed a light for future studies of acupuncture for MUI that placebo/sham acupuncture as control group should be considered to provide more data on net effect of acupuncture.

The study has several limitations. First, the number of studies retrieved and included in this review was very limited. And varied levels of risk of bias and heterogeneity found across studies on proportion of patients with various types of MUIs, treatment regimen, outcome measures selection and sample size etc made it impossible to synthesize the result of individual study. As a result, we cannot provide an overall estimation of the effect size of acupuncture on MUI. Secondly, due to the limited information on baseline characteristics and outcome data collected from the three studies, it was impossible to weigh out the effect size of acupuncture on either urgent or stress component of MUI. In addition, the review only focused on female patients due to the overwhelming incidence of MUI in women compared with men, still it could hinder the generalizability of the result to male population.

## Conclusion

Although acupuncture showed some benefit on reducing the amount of urine leakage and number of incontinence episodes for women with MUI, more evidences were required to draw a solid conclusion of effectiveness and safety of acupuncture among women and even broader population with MUI. Furthermore, it is necessary to optimize study design (i.e., using placebo/sham acupuncture/no treatment as control group), standardize the outcome measurement and use of EA and MA, etc. in order to generate more rigorous evidence to guide the clinical practice in the future.

## Data Availability Statement

The original contributions presented in the study are included in the article/supplementary material, further inquiries can be directed to the corresponding author/s.

## Author Contributions

ZLi: conceptualization. ZLo, HC, and SY: study selection and data extraction. XW and SY: software and analysis. ZLo, HC, and ZLi: writing. All authors have read and agreed to the published version of the manuscript.

## Funding

This study was funded by 2019 National Administration of Traditional Chinese Project of Building Evidence Based Practice Capacity for TCM-Project BEBPC-TCM (No. 2019XZZX-ZJ) and the Fundamental Research Funds for the Central Public Welfare Research Institutes (ZZ13-024-9).

## Conflict of Interest

The authors declare that the research was conducted in the absence of any commercial or financial relationships that could be construed as a potential conflict of interest.

## Publisher's Note

All claims expressed in this article are solely those of the authors and do not necessarily represent those of their affiliated organizations, or those of the publisher, the editors and the reviewers. Any product that may be evaluated in this article, or claim that may be made by its manufacturer, is not guaranteed or endorsed by the publisher.

## References

[1] Myers DL. Female mixed urinary incontinence: a clinical review. JAMA. (2014) 311:2007–14. 10.1001/jama.2014.4299 24846038

[2] Bandukwala NQ Gousse AE. Mixed urinary incontinence: what first? Curr Urol Rep. (2015) 16:9. 10.1007/s11934-015-0483-0 25677232

[3] Dooley Y Lowenstein L Kenton K FitzGerald M Brubaker L. Mixed incontinence is more bothersome than pure incontinence subtypes. Int Urogynecol J Pelvic Floor Dysfunct. (2008) 19:1359–62. 10.1007/s00192-008-0637-4 18491026

[4] Irwin DE Kopp ZS Agatep B Milsom I Abrams P. Worldwide prevalence estimates of lower urinary tract symptoms, overactive bladder, urinary incontinence and bladder outlet obstruction. BJU Int. (2011) 108:1132–8. 10.1111/j.1464-410X.2010.09993.x 21231991

[5] Xue K Palmer MH Zhou F. Prevalence and associated factors of urinary incontinence in women living in China: a literature review. BMC Urol. (2020) 20:159. 10.1186/s12894-020-00735-x 33054777 PMC7559450

[6] Abufaraj M Xu T Cao C Siyam A Isleem U Massad A . Prevalence and trends in urinary incontinence among women in the United States, 2005-2018. Am J Obstet Gynecol. (2021) 225:161–6. 10.1016/j.ajog.2021.03.016 33727114

[7] Mourad S Shokeir A Ayoub N Ibrahim M Reynolds N Donde S . Prevalence and impact of lower urinary tract symptoms: results of the epic survey in Egypt. Neurourol Urodyn. (2019) 38:637–43. 10.1002/nau.23875 30575129

[8] Yang B Foley S. Overview on the management of adult urinary incontinence. Surgery. (2020) 38:204–11. 10.1016/j.mpsur.2020.01.016

[9] Grimshaw R Jain P Latthe P. Management of mixed urinary incontinence. Womens Health. (2012) 8:567–77. 10.2217/whe.12.38 22934730

[10] Chughtai B Laor L Dunphy C Lee R Te A Kaplan S. Diagnosis, evaluation, and treatment of mixed urinary incontinence in women. Rev Urol. (2015) 17:78–83. 10.3909/riu0653 27222643 PMC4857898

[11] Schimpf MO Patel M O'Sullivan DM Tulikangas PK. Difference in quality of life in women with urge urinary incontinence compared to women with stress urinary incontinence. Int Urogynecol J Pelvic Floor Dysfunct. (2009) 20:781–6. 10.1007/s00192-009-0855-4 19495539

[12] Abrams P Andersson KE Birder L Brubaker L Cardozo L Chapple C . Fourth International consultation on incontinence recommendations of the international scientific committee: evaluation and treatment of urinary incontinence, pelvic organ prolapse, and fecal incontinence. Neurourol Urodyn. (2010) 29:213–40. 10.1002/nau.20870 20025020

[13] Campbell SE Glazener CM Hunter KF Cody JD Moore KN. Conservative management for postprostatectomy urinary incontinence. Cochrane Database Syst Rev. (2012) 1:D1843. 10.1002/14651858.CD001843.pub4 22258946

[14] Nambiar AK Bosch R Cruz F Lemack GE Thiruchelvam N Tubaro A . EAU Guidelines on assessment and nonsurgical management of urinary incontinence. Eur Urol. (2018) 73:596–609. 10.1016/j.eururo.2017.12.031 29398262

[15] Khullar V Hill S Laval KU Schiøtz HA Jonas U Versi E. Treatment of urge-predominant mixed urinary incontinence with tolterodine extended release: a randomized, placebo-controlled trial. Urology. (2004) 64:269–74, 274–5. 10.1016/j.urology.2004.02.029 15302476

[16] Özkidik M Coşkun A Asutay MK Bahçeci T Hamidi N. Efficacy and tolerability of mirabegron in female patients with overactive bladder symptoms after surgical treatment for stress urinary incontinence. Int Braz J Urol. (2019) 45:782–9. 10.1590/S1677-5538.IBJU.2018.0518 31136113 PMC6837616

[17] Li J Yang L Pu C Tang Y Yun H Han P. The role of duloxetine in stress urinary incontinence: a systematic review and meta-analysis. Int Urol Nephrol. (2013) 45:679–86. 10.1007/s11255-013-0410-6 23504618

[18] Welk B Baverstock RJ. The management of mixed urinary incontinence in women. Can Urol Assoc J. (2017) 11:S121–4. 10.5489/cuaj.4584 28616109 PMC5461142

[19] Zhong Y Song Y Zeng F Zhao Y Black B Guan Y. Effectiveness of electroacupuncture for female stress urinary incontinence: a systematic review and Meta-analysis. J Tradit Chin Med. (2020) 40:707–20. 10.19852/j.cnki.jtcm.2020.05.001 33000572

[20] Yang N Ge X Ye J Liu Q Wu Y Yan H . Efficacy of acupuncture for urinary incontinence in middle-aged and elderly women: a systematic review and meta-analysis of randomized controlled trials. Eur J Obstet Gynecol Reprod Biol. (2021) 257:138–43. 10.1016/j.ejogrb.2020.11.001 33419589

[21] Qiong W Zhengliang C Jiaqi S Saiqun L Youjun Z Wei Z. Acupuncture for urge urinary incontinence: a systematic review. J Clin Acupun Moxibus. (2015) 31:50–2. 10.3969/j.issn.1005-0779.2015.08.019

[22] Cumpston M Li T Page MJ Chandler J Welch VA Higgins JP . Updated guidance for trusted systematic reviews: a new edition of the Cochrane Handbook for Systematic Reviews of Interventions. Cochrane Database Syst Rev. (2019) 10:D142. 10.1002/14651858.ED000142 31643080 PMC10284251

[23] Engberg S Cohen S Sereika SM. The efficacy of acupuncture in treating urge and mixed incontinence in women: a pilot study. J Wound Ostomy Continence Nurs. (2009) 36:661–70. 10.1097/WON.0b013e3181bd82dd 19920749

[24] Bergström K Carlsson CP Lindholm C Widengren R. Improvement of urge- and mixed-type incontinence after acupuncture treatment among elderly women - a pilot study. J Auton Nerv Syst. (2000) 79:173–80. 10.1016/s0165-1838(99)00077-6 10699649

[25] Liu B Liu Y Qin Z Zhou K Xu H He L . Electroacupuncture versus pelvic floor muscle training plus solifenacin for women with mixed urinary incontinence: a randomized noninferiority trial. Mayo Clin Proc. (2019) 94:54–65. 10.1016/j.mayocp.2018.07.021 30611454

[26] Zhan J Wang X Fei L. Clinical observation on electroacupuncture treatment of mixed urinary incontinence. Health Bull MedSection. (2014) 19:176–7.

[27] Solberg M Alræk T Mdala I Klovning A. A pilot study on the use of acupuncture or pelvic floor muscle training for mixed urinary incontinence. Acupunct Med. (2016) 34:7–13. 10.1136/acupmed-2015-010828 26362793 PMC4789711

[28] Abrams P Cardozo L Fall M Griffiths D Rosier P Ulmsten U . The standardisation of terminology in lower urinary tract function: report from the standardisation sub-committee of the International Continence Society. Urology. (2003) 61:37–49. 10.1016/s0090-4295(02)02243-4 12559262

[29] Lim R Liong ML Lim KK Leong WS Yuen KH. The minimum clinically important difference of the international consultation on incontinence questionnaires (ICIQ-UI SF and ICIQ-LUTSqol). Urology. (2019) 133:91–5. 10.1016/j.urology.2019.08.004 31415780

[30] Karantanis E Allen W Stevermuer TL Simons AM O'Sullivan R Moore KH. The repeatability of the 24-hour pad test. Int Urogynecol J Pelvic Floor Dysfunct. (2005) 16:63–8, 68. 10.1007/s00192-004-1199-8 15647965

[31] de Groat WC Griffiths D Yoshimura N. Neural control of the lower urinary tract. Compr Physiol. (2015) 5:327–96. 10.1002/cphy.c130056 25589273 PMC4480926

[32] Yang L Wang Y Mo Q Liu Z. A comparative study of electroacupuncture at Zhongliao (BL33) and other acupoints for overactive bladder symptoms. Front Med. (2017) 11:129–36. 10.1007/s11684-016-0491-6 28194560

[33] Liu Z Liu Y Xu H He L Chen Y Fu L . Effect of electroacupuncture on urinary leakage among women with stress urinary incontinence: a randomized clinical trial. JAMA. (2017) 317:2493–501. 10.1001/jama.2017.7220 28655016 PMC5815072

[34] Paik SH Han SR Kwon OJ Ahn YM Lee BC Ahn SY. Acupuncture for the treatment of urinary incontinence: a review of randomized controlled trials. Exp Ther Med. (2013) 6:773–80. 10.3892/etm.2013.1210 24137264 PMC3786848

[35] Ferreira CH Bø K. The pad test for urinary incontinence in women. J Physiother. (2015) 61:98. 10.1016/j.jphys.2014.12.001 25744851

[36] Price DM Noblett K. Comparison of the cough stress test and 24-h pad test in the assessment of stress urinary incontinence. Int Urogynecol J. (2012) 23:429–33. 10.1007/s00192-011-1602-1 22086265

[37] Jimenez-Cidre MA Lopez-Fando L Esteban-Fuertes M Prieto-Chaparro L Llorens-Martinez FJ Salinas-Casado J . The 3-day bladder diary is a feasible, reliable and valid tool to evaluate the lower urinary tract symptoms in women. Neurourol Urodyn. (2015) 34:128–32. 10.1002/nau.22530 24264859

[38] Avery K Donovan J Peters TJ Shaw C Gotoh M Abrams P . A brief and robust measure for evaluating the symptoms and impact of urinary incontinence. Neurourol Urodyn. (2004) 23:322–30. 10.1002/nau.20041 15227649

[39] Vaughan CP Markland AD. Urinary incontinence in women. Ann Intern Med. (2020) 172:C17–32. 10.7326/AITC202002040 32016335

