# Supplementary material for: Effect of Acupuncture for Mixed Urinary Incontinence in Women: A Systematic Review
Source: Front Public Health. 2022 Mar 18;10:827853. doi: 10.3389/fpubh.2022.827853 (PMC8971660; doi:10.3389/fpubh.2022.827853)
Supplement: Supplementary file 1 [file Data_Sheet_1.docx]

**Appendix A: Search strategy**

| **Pubmed** | |
| --- | --- |
| (((((("Women"[Mesh]) OR "Female"[Mesh]) OR (woman[Title/Abstract])) AND ((( "Urinary Incontinence"[Mesh]) OR ('urine incontinence'[Title/Abstract])) OR (UI[Title/Abstract]))) AND ((((('mixed urinary incontinence'[Title/Abstract]) OR ('mixed UI'[Title/Abstract])) OR ('mixed incontinence'[Title/Abstract])) OR (MUI[Title/Abstract])) OR (((((('mixed type'[Title/Abstract]) OR ('urgen* -predominant'[Title/Abstract])) OR ('stress-predominant'[Title/Abstract])) OR ('stress[Title/Abstract] AND urgen* '[Title/Abstract])) OR ('stress adj3 mixed'[Title/Abstract])) OR ('urgen* adj3 mixed'[Title/Abstract])))) AND ((("Electroacupuncture"[Mesh]) OR "Acupuncture"[Mesh]) OR (((EA[Title/Abstract]) OR (electroacupunct*[Title/Abstract])) OR (acupunct*[Title/Abstract]) OR 'auricular acupuncture'[Title/Abstract] OR 'ear acupuncture'[Title/Abstract] OR 'scalp acupuncture'[Title/Abstract] OR 'intradermal acupuncture'[Title/Abstract] OR 'dry needle'[Title/Abstract] OR 'dry needling'[Title/Abstract] OR 'fire needle'[Title/Abstract] OR 'fire needling'[Title/Abstract] OR 'abdominal acupuncture'[Title/Abstract] OR 'elongated needle'[Title/Abstract]))) AND ((((((((((((('randomized controlled trial* ') ) OR (RCT)) OR (RCTs)) OR ('quasi-randomized controlled trial* ')) OR ('quasi-RCT* ')) OR ('controlled clinical trial* ')) OR (random*)) OR (trial*)) OR ('sham treatment* ')) OR ('sham acupunctu* ')) OR ('sham EA')) OR ('sham electroacupunct* ')) | 12 |

| **Embase** | |
| --- | --- |
| ('female'/exp OR women:ab,ti OR woman:ab,ti) AND ('urine incontinence'/exp OR 'urinary incontinence':ab,ti OR 'ui':ab,ti) AND ('mixed incontinence'/exp OR 'mixed urinary incontinence':ab,ti OR 'mixed ui':ab,ti OR mui:ab,ti OR 'mixed type':ab,ti OR 'urgen* -predominant':ab,ti OR 'stress-predominant':ab,ti OR 'stress and urgen*':ab,ti OR 'stress adj3 mixed':ab,ti OR 'urgen* adj3 mixed':ab,ti) AND ('electroacupuncture'/exp OR 'acupuncture'/exp OR ea:ab,ti OR electroacupunct*:ab,ti OR acupunct*:ab,ti OR 'auricular acupuncture':ab,ti OR 'ear acupuncture':ab,ti OR 'scalp acupuncture':ab,ti OR 'intradermal acupuncture':ab,ti OR 'dry needle':ab,ti OR 'dry needling':ab,ti OR 'fire needle':ab,ti OR 'fire needling':ab,ti OR 'abdominal acupuncture':ab,ti OR 'elongated needle':ab,ti) AND ('randomized controlled trial'/exp OR 'randomized controlled trial':ab,ti OR rct:ab,ti OR rcts:ab,ti OR 'quasi-randomized controlled trial*':ab,ti OR 'quasi-rct*':ab,ti OR 'controlled clinical trial*':ab,ti OR random*:ab,ti OR trial*:ab,ti OR 'sham treatment*':ab,ti OR 'sham acupunctu*':ab,ti OR 'sham ea':ab,ti OR 'sham electroacupunct*':ab,ti) | 14 |

| **Web of science** | | |
| --- | --- | --- |
| #1 | TS=(female)OR TS=(women)OR TS=(woman) | 2468094 |
| #2 | TS=('urine incontinence')OR TS=('urinary incontinence')OR TS=(UI) | 47434 |
| #3 | #1 AND #2 | 21068 |
| #4 | TS=('mixed incontinence') OR TS=('mixed urinary incontinence') OR TS=('mixed ui') OR TS=('MUI') OR TS=('mixed type') OR TS=('urgen* -predominant') OR TS=('stress-predominant') OR TS=('stress and urgen* ') OR TS=('stress adj3 mixed') OR TS= ('urgen* adj3 mixed') | 201536 |
| #5 | #3 AND #4 | 2343 |
| #6 | TS=(electroacupunct*) OR TS=(acupunct*) OR TS=(EA) OR TS=('auricular acupuncture') OR TS=('ear acupuncture') OR TS=('scalp acupuncture') OR TS=(' intradermal acupuncture') OR TS=('dry needle') OR TS=('dry needling') OR TS=('fire needle') OR TS=('fire needling') OR TS=('abdominal acupuncture') OR TS=('elongated needle') | 65839 |
| #7 | #5 AND #6 | 28 |
| #8 | ALL=('randomized controlled trial*')OR ALL=(RCT) OR ALL=(RCTs)OR ALL=('quasi-randomized controlled trial*') OR ALL=('quasi-rct*') OR ALL=('controlled clinical trial*')OR ALL=(random*) OR ALL=(trial*) OR ALL=('sham treatment*') OR ALL=('sham acupunctu*') OR ALL=('sham EA') OR ALL=('sham electroacupunct*') | 3123717 |
| #9 | #7 AND #8 | 25 |

| **Scopus** | |
| --- | --- |
| ((((TITLE-ABS-KEY (female) OR TITLE-ABS-KEY (women) OR TITLE-ABS-KEY (woman)) AND  (TITLE-ABS-KEY ('urine AND incontinence') OR TITLE-ABS-KEY ('urinary AND incontinence') OR  TITLE-ABS-KEY(ui))) AND (TITLE-ABS-KEY ('mixed AND incontinence') OR TITLE-ABS-KEY  ('mixed AND urinary AND incontinence') OR TITLE-ABS-KEY('mixed AND ui') OR  TITLE-ABS-KEY ('mui') OR TITLE-ABS-KEY ( 'mixed AND type') OR TITLE-ABS-KEY  ('urgen* AND -predominant') OR TITLE-ABS-KEY ('stress-predominant') OR TITLE-ABS-KEY  ('stress  AND  urgen*') OR TITLE-ABS-KEY ('stress AND adj3 AND mixed' ) OR TITLE-ABS-KEY ('urgen* AND adj3 AND mixed'))) AND (TITLE-ABS-KEY (electroacupunct*) OR TITLE-ABS-KEY  (acupunct*) OR TITLE-ABS-KEY (ea) OR TITLE-ABS-KEY ('auricular AND acupuncture') OR  TITLE-ABS-KEY ('ear AND acupuncture') OR TITLE-ABS-KEY ('scalp AND acupuncture') OR  TITLE-ABS-KEY ('intradermal AND acupuncture') OR TITLE-ABS-KEY ('dry AND needle') OR  TITLE-ABS-KEY ('dry AND needling') OR TITLE-ABS-KEY ('fire AND needle') OR  TITLE-ABS-KEY ('fire AND needling') OR TITLE-ABS-KEY ('abdominal AND acupuncture') OR  TITLE-ABS-KEY ('elongated AND needle'))) AND (ALL('randomized AND controlled AND trial*')  OR ALL (rct) OR ALL (rcts) OR ALL ('quasi-randomized AND controlled AND trial*') OR ALL  ('quasi-rct*') OR ALL ('controlled AND clinical AND trial*') OR ALL (random*) OR ALL (trial*) OR  ALL ('sham AND treatment*') OR ALL ('sham AND acupunctu*') OR ALL('sham AND ea')  OR ALL('sham AND electroacupunct*')) | 36 |

| **Cochrane Library** | | |
| --- | --- | --- |
| #1 | MeSH descriptor: [Female] in all MeSH products | 463244 |
| #2 | MeSH descriptor: [Women] explode all trees | 695 |
| #3 | (woman):ti,ab,kw | 158978 |
| #4 | #1 OR #2 OR #3 | 555666 |
| #5 | ('urinary incontinence'):ti,ab,kw OR ('urine incontinence'):ti,ab,kw OR (UI):ti,ab,kw | 8732 |
| #6 | #4 AND #5 | 4204 |
| #7 | ('mixed urinary incontinence'):ti,ab,kw OR ('mixed incontinence'):ti,ab,kw OR (MUI):ti,ab,kw OR ('mixed type'):ti,ab,kw OR ("urgen* -predominant"):ti,ab,kw | 7781 |
| #8 | ('stress-predominant'):ti,ab,kw OR ('stress and urgen*'):ti,ab,kw OR ('stress adj3 mixed'):ti,ab,kw OR ('urgen* adj3 mixed'):ti,ab,kw | 788 |
| #9 | #7 OR #8 | 8391 |
| #10 | #6 AND #9 | 702 |
| #11 | MeSH descriptor: [Electroacupuncture] explode all trees | 841 |
| #12 | MeSH descriptor: [Acupuncture] explode all trees | 155 |
| #13 | (EA):ti,ab,kw OR (electroacupunct*):ti,ab,kw OR (acupunct*):ti,ab,kw OR ('auricular acupuncture'):ti,ab,kw OR ('ear acupuncture'):ti,ab,kw | 18614 |
| #14 | ('scalp acupuncture'):ti,ab,kw OR ('intradermal acupuncture'):ti,ab,kw OR ('dry needle'):ti,ab,kw OR ('dry needling'):ti,ab,kw OR ('fire needle'):ti,ab,kw | 1527 |
| #15 | ('fire needling'):ti,ab,kw OR ('abdominal acupuncture'):ti,ab,kw OR ('elongated needle'):ti,ab,kw | 713 |
| #16 | #13 OR #14 OR #15 | 19475 |
| #17 | #10 AND #16 | 25 |
| #18 | MeSH descriptor: [Randomized Controlled Trial] explode all trees | 119 |
| #19 | ('randomized controlled trial*') OR (RCT) OR (RCTs) OR ('quasi-randomized controlled trial*') OR ('quasi-rct*') | 1197897 |
| #20 | ('controlled clinical trial*') OR (random*) OR (trial*) OR ('sham treatment*') OR ('sham acupunctu*') | 1797918 |
| #21 | ('sham EA'):ti,ab,kw OR ('sham electroacupunct*'):ti,ab,kw | 530 |
| #22 | #19 OR #20 OR #21 | 1798117 |
| #23 | #17 AND #22 | 22 |

| **CBM** | |
| --- | --- |
| ("random" OR "controlled" OR "clinical" OR "trial" OR "study" OR "observational" OR "sham electroacupuncture" OR "sham acupuncture") AND (("electroacupuncture" OR "acupuncture" OR "auricular acupuncture" OR "scalp acupuncture" OR "intradermal acupuncture" OR "dry needle" OR "fire needle" OR "abdominal acupuncture" OR "elongated needle") AND (("'mixed" OR "stress-predominant" OR "urge-predominant") AND ("female" AND "urinary incontinence"))) | 6 |

| **CNKI** | |
| --- | --- |
| (SU %= 'female' AND SU %= 'urinary incontinence') AND (SU %= 'mixed' OR SU %= 'stress-predominant' OR SU %= 'urge-predominant') AND (SU %= 'electroacupuncture' OR SU %= 'acupuncture' OR SU %= 'auricular acupuncture' OR SU %= 'scalp acupuncture' OR SU %= 'intradermal acupuncture' OR SU %= 'dry needle' OR SU %= 'fire needle' OR SU %= 'abdominal acupuncture' OR SU %= 'elongated needle') AND (FT %= 'random' OR FT %= 'controlled' OR FT %= 'trial' OR FT %= 'clinical' OR FT %= 'study' OR FT %= 'observational' OR FT %= 'sham electroacupunctur' OR FT %= 'sham acupuncture') | 20 |

| **VIP** | |
| --- | --- |
| M=female AND M=urinary incontinence AND (M=(mixed OR stress-predominant OR urge-predominant)) AND (M=(electroacupuncture OR acupuncture OR auricular acupuncture OR scalp acupuncture OR intradermal acupuncture OR dry needle OR fire needle OR abdominal acupuncture OR elongated needle)) AND (U=(random OR controlled OR trial OR clinical OR study OR observational OR sham electroacupuncture OR sham acupuncture)) | 5 |

| **VANFANG DATA** | |
| --- | --- |
| Subject::("female" and "urinary incontinence") and Subject:("mixed" OR "stress-predominant" OR "urge-predominant") and Subject::("electroacupuncture" OR "acupuncture" OR "auricular acupuncture" OR "scalp acupuncture" OR "intradermal acupuncture" OR "dry needle" OR "fire needle" OR "abdominal acupuncture" OR "elongated needle") and ALL:("random" OR "controlled" OR "trial" OR "clinical" OR "study" OR "observational" OR "sham electroacupuncture" OR "sham acupuncture" ) | 11 |

| **Clinical.tral** | |
| --- | --- |
| “mixed urinary incontinence” female with results | 6 |

**Appendix B: Characteristics of excluded studies [ordered by study ID]**

| Study | Reason for exclusion |
| --- | --- |
| Zhang et al 2014 | No outcome data available |
| Sun et al 2018 | No outcome data available |
| Sun et al 2020 | No outcome data available |
| Silvia et al 2021 | No outcome data available |
| Bergström et al 2000 | No outcome data available |
| Engberg et al 2009 | No outcome data available |
| Engberg et al 2010 | Conference Abstract |
| Liu et al 2019 | Conference Abstract |
| Pang et al 2012 | Duplication with other studies |
| Shi et al 2015 | Duplication with other studies |
| Zhang et al 2015 | Duplication with other studies |
| Zhang et al 2015 | Duplication with other studies |
| Chen et al 2016 | Duplication with other studies |
| Wang et al 2016 | Duplication with other studies |
| Wang et al 2016 | Duplication with other studies |
| Chen et al 2016 | Duplication with other studies |
| Chen et al 2016 | Duplication with other studies |
| Chen et al 2016 | Duplication with other studies |
| Wang et al 2017 | Duplication with other studies |
| Jin et al 2014 | Meet the exclusion criteria |
| Zhan et al 2016 | Meet the exclusion criteria |
| Pang et al 2016 | Meet the exclusion criteria |
| Jin et al 2013 | Not RCT |
